# CoolCuddle and Autonomic Regulation in Infants With Hypoxic‐Ischaemic Encephalopathy: An Intervention Study

**DOI:** 10.1111/apa.70547

**Published:** 2026-04-16

**Authors:** Ela Chakkarapani, Satomi Okano, David Odd, CoolCuddle Study Investigators

**Affiliations:** ^1^ Centre for Academic Child Health, Bristol Medical School University of Bristol Bristol UK; ^2^ Neonatal Intensive Care Unit, St Michael's Hospital University Hospitals Bristol & Weston NHS Trust Bristol UK; ^3^ Division of Population Medicine Cardiff University School of Medicine Cardiff UK

**Keywords:** CoolCuddle, heart rate variability, hypoxic‐ischaemic encephalopathy, therapeutic hypothermia

## Abstract

**Aim:**

We evaluated the impact of ‘CoolCuddle’, parental cuddling during therapeutic hypothermia intervention for hypoxic‐ischaemic encephalopathy, on heart rate variability (HRV).

**Methods:**

In this prospective, single‐group, interventional study with repeated measures, we included infants ≥ 36 weeks' gestation undergoing hypothermia and CoolCuddle with HRV data. Time‐domain HRV was assessed before, during, and after each cuddle. Primary outcomes were heart rate, mean RR interval (ANN), standard deviation of normal RR intervals, and root mean square of successive differences (RMSSD).

**Results:**

In 65 CoolCuddles from 26 infants, CoolCuddle did not significantly alter HRV. However, infants with a 1‐min Apgar < 7 had increased heart rates during (+2.33 bpm) and after (+3.27 bpm) cuddling, whereas those with higher Apgar scores showed decreases (−1.43 and −8.21 bpm) compared with pre‐cuddle. Infants with Apgar < 7 or abnormal aEEG had reduced ANN during (−11.96 ms, −12.75 ms) and after (−19.89 ms, −19.00 ms), while those with higher Apgar or normal aEEG showed increases (+15.32 ms, +23.33 ms(during‐cuddle); +63.94 ms, +55.06 ms (post‐cuddle)). Maternal cuddling was associated with higher post‐cuddle RMSSD (+1.06 ms) than paternal cuddling (−0.73 ms), (*p* = 0.04).

**Conclusion:**

While CoolCuddle had little overall effect on HRV, autonomic responses varied by asphyxia and encephalopathy severity and parent, suggesting differential modulation in vulnerable infants.

## Summary

- Parent‐newborn skin‐to‐skin contact may influence heart rate variability (HRV), but the effects of cuddling infants with hypoxic‐ischaemic encephalopathy receiving cooling therapy on heart rate variability were unclear.
- Overall, HRV was unchanged; however, autonomic responses varied by asphyxia and encephalopathy severity, with differential changes in heart rate and mean RR interval (ANN) and higher post‐cuddle root mean square of differences (RMSSD) after maternal cuddling.
- Supports safety and individualised physiological monitoring during CoolCuddle.

**Abbreviations**
aEEG: amplitude‐integrated electroencephalogram
ANN: mean normal‐to‐normal interval milliseconds (ms)
bpm: beats per minute
HIE: hypoxic–ischaemic encephalopathy
HRV: heart rate variability
IQR: interquartile range
ISRCTN: International Standard Randomised Controlled Trial Number
ms: milliseconds
NICU: neonatal intensive care unit
NIHR: National Institute for Health and Care Research
NN50: number of successive normal‐to‐normal intervals differing by > 50 milliseconds
pNN50: proportion of successive normal‐to‐normal intervals differing by > 50 milliseconds
RMSSD: root mean square of successive normal‐to‐normal interval differences, milliseconds
SD: standard deviation
SDNN: standard deviation of all normal‐to‐normal R‐R (NN) intervals
SE: standard error
UK: United Kingdom

## 1 Introduction

Hypoxic‐ischaemic encephalopathy (HIE) due to birth asphyxia affects 1–3/1000 live births in high‐income countries [1]. HIE is a major cause of death and lifelong disability. Globally, HIE is the 2nd leading contributor to disability‐adjusted life years among neurological disorders [2]. Therapeutic hypothermia improves survival and reduces disability, making it the standard of care in high‐income settings [3].

However, despite therapeutic hypothermia, many children experience cognitive difficulties, with an average intelligence quotient deficit of 14 points, attention and communication problems, even in the absence of motor disabilities [4, 5, 6, 7]. Strengthening parent‐infant bonding may promote cognitive development. Early physical and emotional interaction, such as skin‐to‐skin contact, supports bonding and autonomic regulation [8, 9]. To enable such interaction during therapeutic hypothermia, we developed ‘CoolCuddle’, a structured nurse‐led procedure that allows parents to cuddle their infants during therapeutic hypothermia safely [10, 11].

CoolCuddle improves parent‐infant bonding and has minimal effects on rectal temperature, oxygen saturation, or the quality and pattern of the amplitude‐integrated electroencephalogram (aEEG) [10, 11, 12]. However, its impact or interaction on the infant's autonomic regulation is unknown.

HIE affects the autonomic nervous system [13, 14], and the severity of HIE is associated with autonomic nervous system dysfunction, as measured by heart rate variability (HRV) [15]. Similar disturbances in autonomic nervous system regulation occur in preterm infants [16], where skin‐to‐skin contact improves HRV, suggesting improved autonomic nervous system regulation and maturation [17]. Although CoolCuddle does not involve complete skin‐to‐skin contact, continuous physical and emotional interaction may influence autonomic nervous system regulation. Factors such as asphyxia severity [18], encephalopathy grade [15] and demographic characteristics [19] could also modulate this effect.

Therefore, we investigated whether the CoolCuddle intervention would alter HRV measures and whether HRV measures would be affected by infant characteristics, asphyxia severity and encephalopathy grade. Our objectives were:

1. To determine whether the CoolCuddle intervention alters HRV measures by assessing differences in HRV measures between the pre‐cuddle, during‐cuddle and post‐cuddle periods and quantifying the effect of cuddle on HRV measures compared with the pre‐cuddle.
2. To examine associations between baseline HRV (pre‐cuddle period) and infant characteristics, asphyxia severity and encephalopathy grade.
3. To assess whether the effect of CoolCuddle intervention on HRV differs according to infant characteristics, asphyxia severity and encephalopathy grade.

## 2 Methods

### 2.1 Study Design and Setting

This prospective, single‐group, interventional study with repeated measures was conducted between October 2019 and November 2020 in two tertiary neonatal intensive care units (NICUs), St Michael's Hospital and Southmead Hospital, Bristol, UK. Detailed methods have been reported previously [10].

### 2.2 Participants

We approached parents for consent to CoolCuddle if their infants were born at ≥ 36 weeks of gestation, undergoing therapeutic hypothermia using a servo‐controlled cooling device and intensive care for HIE. We excluded infants who received significant cardiorespiratory support (one or more of: high‐frequency oscillation, mean airway pressure > 12 cmH_2_O, inhaled nitric oxide, oxygen > 70%, > 1 chest drain, or ≥ 3 inotropes), congenital anomalies, status epilepticus before cuddle or insufficient English to complete bonding/mental health questionnaires [20]. CoolCuddle followed a standard operating procedure: two trained nurses completed safety checks, prepared the infant and assisted with positioning; an advanced neonatal nurse practitioner supervised the process. Each CoolCuddle session comprised a 60‐min pre‐cuddle observation, up to 2 h of cuddling and a 60‐min post‐cuddle observation.

### 2.3 HRV Measures

HRV was recorded using the VitaLogik 6000 series monitor (Mennen Medical) in 5‐min epochs via standard three‐lead ECG. The algorithm identified R‐wave peaks to compute normal‐to‐normal intervals, excluding ectopic beats. The following time‐domain HRV indices were analysed:

- ANN: mean normal‐to‐normal interval milliseconds (ms)
- SDNN: standard deviation of normal‐to‐normal intervals of normal beats reflecting overall HRV influenced by both sympathetic and parasympathetic tone, milliseconds (ms) [21].
- RMSSD: root mean square of successive normal‐to‐normal interval differences, reflecting parasympathetic activity, milliseconds (ms) [22].
- NN50 and pNN50: number and proportion of successive normal‐to‐normal intervals differing by > 50 ms, indexing high‐frequency variability [23].

Data acquired during the transition from pre‐cuddle to cuddle or from cuddle to post‐cuddle were not included in the analysis.

### 2.4 Clinical Data

Data on the demographic characteristics, severity of asphyxia and encephalopathy, and doses of morphine and inotropic support were collected from the medical records. Apgar score < 7 was defined as a low Apgar score. Mild encephalopathy was defined as clinical encephalopathy with a normal aEEG. Moderate to severe encephalopathy was defined as moderate to severe abnormalities on aEEG. aEEG was categorised as normal or abnormal (moderate‐to‐severe abnormalities). All infants underwent brain MRI within 10 days after birth. Lesions were scored on the deep grey matter, cortex, white matter and brainstem [24].

### 2.5 Outcomes

Primary outcomes included the heart rate, ANN, SDNN and RMSSD. Secondary outcomes included NN50 and pNN50.

### 2.6 Sample Size

The sample size was chosen pragmatically based on a conservative estimate of the number of parents likely to consent to CoolCuddle, given time and staff availability constraints. In 2017–2018, before the commencement of the study in 2019, on average, 62 eligible infants were cared for per year. To investigate the effect of the CoolCuddle intervention on hypothermia treatment, measured using core temperature and the intensive care, measured using cardiorespiratory and neurophysiology, a convenience sample of between 24 and 30 infants was considered sufficient for a feasibility study, which will be between 39% and 48% recruitment rate and deemed achievable within the funded study period.

### 2.7 Statistical Analysis

Initially, we described the patient demographics, then derived marginal mean (SE) values and distributions for the primary outcomes—ANN, SDNN and RMSSD—split by infant, asphyxia, encephalopathy and cuddle characteristics at the baseline (pre‐cuddle period). Marginal mean values (and SE) were derived using a linear mixed‐effects model, with the outcome and demographic measure as fixed effects and cuddle period, cuddle number and patient ID as (increasingly clustered) random effects.

Characteristics of the infants included sex, gestation, birth weight and measures of asphyxia, including cord blood gas and Apgar scores and severity of encephalopathy, including the grade of HIE and pattern of aEEG before commencing therapeutic hypothermia and cuddle characteristics described by the age at cuddle and whether cuddled by mother or father. All the characteristics were dichotomised as follows: Sex (Male/Female), Gestation (< 40/≥ 40), Birth weight (≤ 3.5 kg/> 3.5 kg), pH (< 7/≥ 7), Apgar (< 7/> 6), HIE grade (Mild/Moderate–severe), aEEG (normal/abnormal pattern) and age at cuddle (< 59/≥ 59 h). Because SDNN, RMSSD, NN50 and pNN50 were clearly skewed, data were log‐transformed for analysis and testing. Measures were back‐transformed to the original distribution to aid interpretation using the Stata ‘nlcom’ command (adjusted using Duan's smearing estimator [25]), while standard errors were derived using the delta method.

The marginal means for each epoch were derived for the primary and secondary outcomes across CoolCuddle (over time) using a multilevel, clustered linear model (infant as the highest level, then cuddle), as per our previous work [10]. Summary measures were derived using a linear mixed effects model, with the outcome and cuddle period in the fixed effects, and cuddle period, cuddle number and patient ID as (increasingly clustered) random effects. Means and standard deviations were derived from the multi‐level models (one per outcome) to account for dependent data, and clustering by infant, cuddle and period of cuddle. Models were compared to one without cuddle‐period information using the likelihood ratio test to assess whether there was evidence of overall differences in the measures across the three periods: pre‐cuddle, cuddle and post‐cuddle. Mean differences between the cuddle and post‐cuddle periods and the pre‐cuddle period were also derived from the above model (along with 95% confidence intervals).

Finally, we tested whether differences in the primary outcomes across the three periods (pre‐cuddle, during‐cuddle and post‐cuddle) varied according to infant, asphyxia, encephalopathy and cuddle characteristics described above. Summary measures were derived using a linear mixed effects model, with the outcome, cuddle period and patient characteristics in the fixed effects, and cuddle period, cuddle number and patient ID as (increasingly clustered) random effects. We also included intensive care measures, including the use of inotropes during the cuddle, and whether the cuddle occurred during the rewarming period, and dichotomised these as inotropes (no/yes) and cuddles during rewarming (no/yes). Models were compared, including these terms as interaction terms with the time periods, and the likelihood ratio test was used to assess the difference.

Results are presented as arithmetic mean (SD), median (IQR), *n*(%), marginal means (standard error (SE)) or marginal mean difference (95% CI) as appropriate. Analysis was performed using Stata 18. A *p* < 0.05 was considered the level of statistical significance.

### 2.8 Ethics Approval

The study was approved by the Research Ethics Committee (reference 19/NI/0143), the UK Health Regulatory Agency (IRAS ID 257430) and registered with ISRCTN 10198406.

## 3 Results

In the CoolCuddle study, 27 infants were recruited, and they underwent 70 CoolCuddles. One infant with no HRV data for the single CoolCuddle they underwent was excluded. Another infant lacked HRV data for two of the three CoolCuddles. Two infants had incomplete HRV data for two of their CoolCuddles, which were excluded, leaving 65/70 CoolCuddles with HRV data. Hence, 26 infants with complete HRV measures recorded across 65 cuddles, including 12 during rewarming, were included in the analysis. Median (IQR) of cuddles per infant was 3 (2,3) with a range of (1,4). We excluded mean HRV data for 31.7 min during the transition from the open crib to the parent and for 21.5 min during the transition from the parent to the open crib across all the included 65 CoolCuddles from 26 infants. The final HRV dataset included 58.08 h during pre‐cuddle, 101.3 h during cuddle and 57.7 h during post‐cuddle.

The mean (SD) age of the mothers was 30.9 (5.0) years, and 23 (88%) were of white ethnicity (Table 1). The majority of mothers were primiparous (16 (62%)), and 18 (69%) infants were born by caesarean section. A total of 18 (69%) infants were male. All infants were born at term (39.6 (1.5)) weeks of gestation. The mean, cord or lowest pH was 6.99 (0.15); 8 (31%) infants had grade 3 HIE, and 24 (92%) infants had a moderate‐severely abnormal aEEG pattern before commencing therapeutic hypothermia. (Table 1).

**TABLE 1:** Characteristics of the study population.

Measure	*n*	Summary measure
Maternal characteristics		
Age (years)	26	30.9 (5.0)
Race—White	26	23 (88%)
Pregnancy characteristics		
Primiparous	26	16 (62%)
Induction of labour	26	3 (12%)
Pregnancy complications^a^	26	22 (85%)
Intrapartum complications^b^	26	4 (15%)
Lower segment caesarean section (LSCS)	26	18 (69%)
Breech	26	1 (4%)
Paternal characteristics		
Age (years)	24	32.7 (5.0)
Race—White	24	20 (83%)
Infant characteristics		
Sex (Male)	26	
Male		18 (69%)
Female		8 (31%)
Gestation weeks (mean (SD))	26	39.6 (1.5)
Birth weight (g), mean (SD)	26	3338 (460)
Cord or lowest pH	22	6.99 (0.15)
Apgar scores		
1 min	26	3 (1–5)
5 min	26	6 (4–7)
10 min	26	7 (5–8)
HIE Grade	26	
I		3 (12%)
II		15 (58%)
III		8 (31%)
Moderate‐severely abnormal aEEG before TH	26	
No		2 (8%)
Yes		24 (92%)
Age at first Cuddle (h)	21	47.0 (16.2)
Age at Cuddle (any)	65	58.7 (16.2)
Cuddled by	65	
Mum		42 (65%)
Dad		23 (35%)

*Note:* Values are mean (standard deviation), Number (%) or Median (IQR) as appropriate. Cord or lowest pH denotes the lowest pH from cord blood gas or blood gas taken from the infant within 60 min after birth. Continuous data are presented as mean (SD), except for Apgar presented as median (IQR) and the categorical data are presented as *n* (%).

^a^ Pre‐eclampsia, HELLP (haemolysis, elevated liver enzymes and a low platelet count) syndrome, pregnancy‐induced hypertension, antepartum bleed, diabetes, Bell's palsy and polyhydramnios.

^b^ Cord prolapse, uterine rupture, shoulder dystocia, placental abruption, fetal decelerations, fetal bradycardia, prolonged rupture of membranes, reduced fetal movements, meconium‐stained liquor.

The mean (SD) age of the 65 CoolCuddles was 58.7 (16.2) hours of age. Infants received a median (IQR) morphine infusion of 30 micrograms/kg/h (20, 40) for analgesia. Morphine was not used in 5/12 CoolCuddles that occurred during the rewarming. In 22 CoolCuddles, infants received a mean (SD) dopamine infusion of 8.1 micrograms/kg/min (3.8) for inotropic support. Fathers provided 23 of the 65 cuddles. During CoolCuddle, aEEG was normal in 66% of infants and abnormal in 34%. MRI showed white matter lesions in 10 infants, cortical lesions in 4, and a deep grey matter lesion in 1 and none of the infants had brain stem lesions.

**FIGURE 1:**
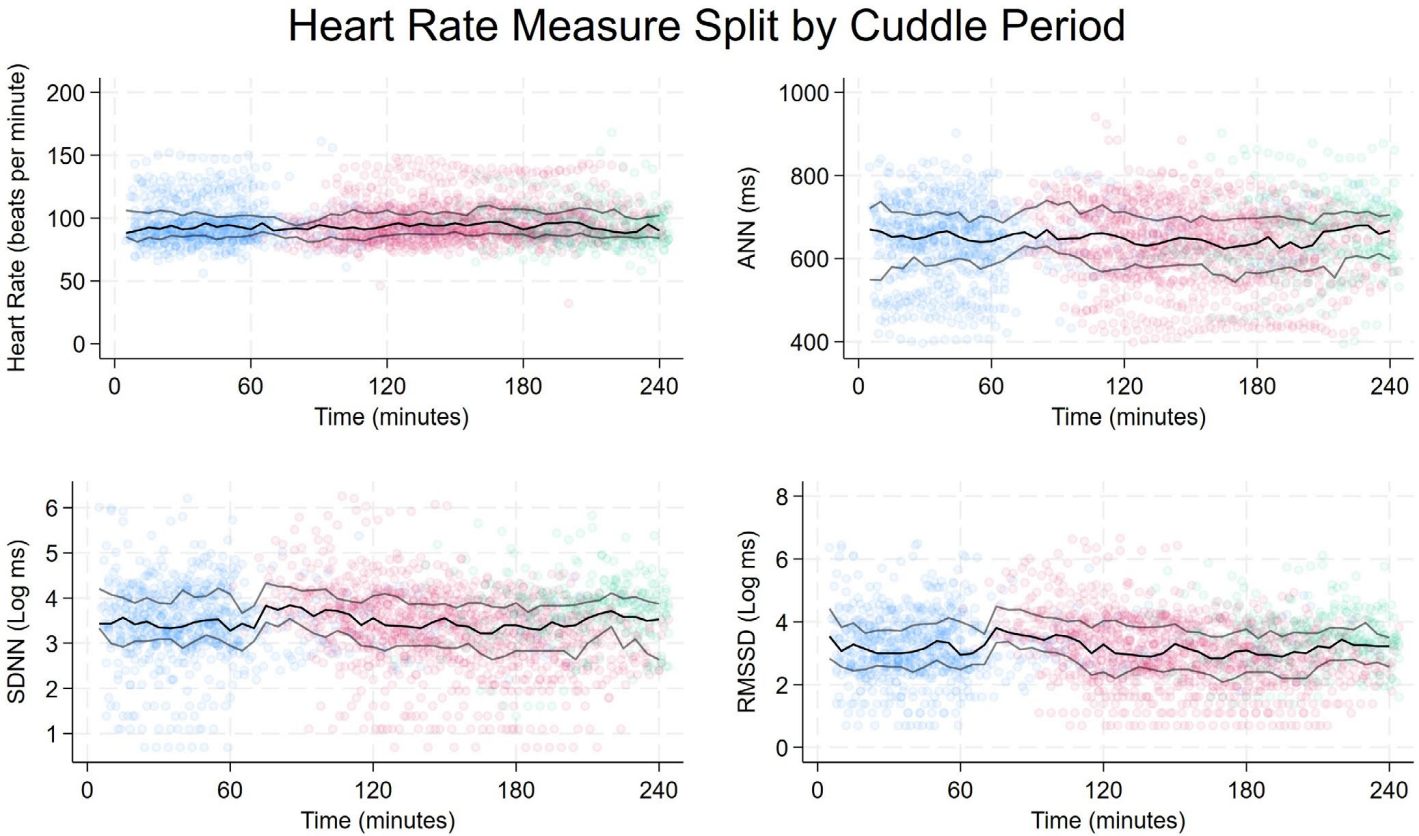
HRV measures split by pre‐cuddle (blue), cuddle (red) and post‐cuddle (green). Heart rate beats per minute; Mean RR interval of normal beats (ANN) milliseconds; Logarithmic conversion of standard deviation of RR intervals of normal beats (SDNN); Logarithmic conversion of root mean square of differences of successive intervals of normal beats (RMSSD). Points represent individual measure of heart rate, or variability. The bold black line is the median measure for that period, alongside the 25th and 75th percentiles. bpm, beats per minute; ms, milliseconds.

At baseline, during the pre‐cuddle period, the primary outcome measures, including ANN, SDNN and RMSSD, were similar across infant characteristics and severity measures of asphyxia or encephalopathy and cuddle characteristics (all *p* > 0.05) (Table 2). Equally, there was little evidence that the primary outcomes, including the heart rate, ANN, SDNN and RMSSD, differed across the CoolCuddle period (all *p* > 0.1) (Table 3, shown in Figure 1). The secondary outcomes, including the NN50 and pNN50, did not differ across the CoolCuddle period (Table S1).

**TABLE 2:** HRV characteristics of the study population at the baseline (pre‐cuddle period).

Infant characteristic	Infants/cuddles	Heart rate, bpm	*p*	ANN ms	*p*	SDNN, ms^a^	*p* ^b^	RMSSD, ms^a^	*p* ^b^
All infants	26/65	96.1 (2.4)		640.4 (14.6)		34.2 (4.2)		34.0 (3.9)	
Sex (male)	26/65		0.99		0.81		0.82		0.80
Female		96.6 (4.4)		642.0 (26.3)		32.8 (7.2)		32.5 (6.7)	
Male		96.7 (2.9)		634.4 (17.5)		34.8 (5.1)		34.6 (4.7)	
Gestation (weeks)	26/65		0.58		0.55		0.18		0.26
< 40		95.6 (3.0)		643.1 (18.1)		38.5 (5.6)		37.4 (5.2)	
≥ 40		98.4 (4.0)		625.0 (24.4)		27.7 (5.5)		28.9 (5.3)	
Birth weight (kg)	26/65		0.63		0.71		0.21		0.34
≤ 3.5 kg		97.7 (3.3)		631.5 (20.0)		29.8 (4.8)		30.8 (4.7)	
> 3.5 kg		95.4 (3.5)		642.5 (21.4)		40.2 (7.0)		38.1 (6.2)	
Cord or lowest (pH)	22/55		0.55		0.46		0.88		0.99
pH < 7		94.6 (2.9)		649.2 (17.7)		37.2 (4.9)		36.4 (4.8)	
pH ≥ 7		97.3 (3.5)		628.4 (21.8)		38.3 (6.1)		36.4 (5.9)	
Apgar scores									
1 min	26/65		0.48		0.29		0.51		0.36
< 7		96.0 (2.5)		642.3 (15.1)		35.2 (4.5)		35.4 (4.1)	
> 6		101.5 (7.3)		593.2 (43.4)		27.2 (9.9)		25.5 (8.7)	
5 min	26/65		0.36		0.58		0.87		0.99
< 7		97.8 (2.7)		632.2 (16.6)		33.8 (4.7)		34.0 (4.4)	
> 6		92.7 (4.8)		651.2 (29.6)		35.4 (8.9)		34.1 (7.8)	
10 min	26/65		0.76		0.95		0.98		0.72
< 7		97.3 (3.3)		637.6 (20.0)		34.3 (5.7)		35.3 (5.4)	
> 6		95.8 (3.6)		635.6 (21.4)		34.1 (6.1)		32.7 (5.4)	
HIE Grade	26/65		0.20		0.18		0.31		0.43
Mild		94.6 (2.8)		649.6 (16.9)		37.1 (5.3)		36.0 (4.9)	
Moderate–severe		101.1 (4.2)		608.7 (25.5)		28.5 (6.2)		29.8 (6.1)	
Initial aEEG pattern	26/65		0.89		0.91		0.91		0.60
Normal pattern		95.7 (7.4)		631.7 (44.4)		32.9 (12.1)		28.7 (10.0)	
Abnormal pattern		96.7 (2.6)		637.3 (15.5)		34.3 (4.4)		34.8 (4.1)	
Age at Cuddle	21/57		0.34		0.32		0.11		0.10
< 59 h		96.8 (3.2)		636.2 (19.2)		29.7 (4.7)		29.0 (4.6)	
≥ 59 h		100.0 (3.1)		618.1 (18.4)		36.2 (5.5)		38.1 (5.8)	
Cuddle by	26/65		0.67		0.78		0.16		0.07
Mum		97.1 (2.6)		635.2 (15.7)		32.8 (4.1)		31.5 (3.8)	
Dad		95.8 (3.2)		640.2 (18.9)		38.5 (5.5)		42.0 (6.4)	

*Note:* Values are marginal means (standard error (SE)). Summary values and statistical tests derived from a multi‐level model accounting for dependent data for infants and cuddles. Cord or lowest pH denotes the lowest pH from cord blood gas or blood gas taken from the infant within 60 min after birth.

^a^ Numbers derived from log‐transformed values, and back‐transformed for presentation.

^b^ *p*‐Value derived from the likelihood ratio test; comparing models with or without the (fixed effect) patient characteristic interaction.

**TABLE 3:** Summary values and mean differences for measures of heart rate variability during the CoolCuddle.

Variable	Infants/cuddles	Pre‐cuddle	During cuddle	Post cuddle	*p* ^a^
Heart rate (bpm)	26/65				
Summary measures		96.1 (2.4)	98.0 (2.4)	98.3 (2.4)	
Mean difference (95% CI)		Ref (0)	1.97 (−0.50 to 4.44)	2.21 (−0.26 to 4.69)	0.16
ANN (ms)	26/65				
Summary measures		640.4 (14.6)	631.0 (14.6)	628.2 (14.6)	
Mean difference (95% CI)		Ref (0)	−9.41 (−26.63 to 7.79)	−12.17 (−29.35 to 5.01)	0.35
SDNN^b^ (ms)	26/65				
Summary measures		33.9 (4.1)	33.3 (4.0)	33.3 (4.0)	
Relative change, % (95% CI)		Ref (0)	−3.68 (−7.37 to 0.02)	−0.55 (−4.37 to 3.27)	0.1
RMSSD^b^ (ms)	26/65				
Summary measures		31.5 (3.9)	27.2 (3.4)	29.6 (3.7)	
Relative change, % (95% CI)		Ref (0)	−4.32 (−8.73 to 0.09)	−1.89 (−6.44 to 2.66)	0.14

*Note:* Values are marginal mean (SE), or marginal mean difference (95% CI) as appropriate. Summary measures and statistical tests, derived from multi‐level model accounting for dependent data for infants and cuddles.

Abbreviations: bpm, beats per minute; ms, milliseconds.

^a^ *p*‐Value derived from the likelihood ratio test; comparing models with or without the (fixed effect) cuddle‐period variable.

^b^ Numbers derived from log‐transformed values, and back‐transformed for presentation.

There was little evidence that HRV measures during the cuddle or post‐cuddle period differed by sex, gestational age, birthweight, cord pH, HIE grade or age of cuddle or rewarming status, compared to the pre‐cuddle period (all *p* > 0.05) (Table 4).

**TABLE 4:** Summary values of heart rate variability changes during the CoolCuddle compared to the pre‐cuddle period, split by patient characteristics.

Measure	Infants/cuddles	Cuddle	Post‐cuddle	*p*_interaction_	Cuddle	Post‐cuddle	*p*_interaction_	Cuddle	Post‐cuddle	*p*_interaction_	Cuddle	Post‐cuddle	*p*_interaction_
		Heart Rate bpm			ANN ms			SDNN^a^ ms			RMSSD^a^ ms		
Infant characteristics													
Sex (male)	26/65			0.35			0.22			0.92			0.90
Female		1.95 (−2.00 to 5.89)	4.52 (0.53 to 8.51)		−14.62 (−41.24 to 11.99)	−33.71 (−60.45 to −6.96)		−2.29 (−6.36 to 1.79)	0.28 (−3.87 to 4.42)		−2.56 (−7.00 to 1.86)	−1.59 (−6.10 to 2.93)	
Male		1.99 (−1.10 to 5.10)	1.19 (−1.90 to 4.27)		−7.12 (−28.70 to 14.45)	−2.57 (−24.05 to 18.90)		−4.36 (−0.55 to 0.82)	−0.98 (−6.36 to 4.39)		−5.18 (−11.53 to 1.16)	−1.97 (−8.54 to 4.61)	
Gestation (weeks)	26/65			0.60			0.95			0.70			0.68
< 40		2.78 (−0.72 to 6.29)	3.08 (−0.43 to 6.59)		−11.48 (−36.41 to 13.46)	−13.64 (−38.45 to 11.17)		−5.39 (−10.65 to −0.12)	−1.87 (−7.27 to 3.53)		−6.10 (−12.39 to 0.19)	−3.74 (−10.16 to 2.67)	
≥ 40		0.59 (−2.39 to 3.57)	0.76 (−2.24 to 3.76)		−5.83 (−25.17 to 13.52)	−9.74 (−29.15 to 0.68)		−1.56 (−6.26 to 3.14)	1.01 (−3.96 to 5.97)		−2.00 (−7.61 to 3.61)	0.47 (−5.44 to 6.39)	
Birth weight (kg)	26/65			0.69			0.40			0.28			0.67
≤ 3.5 kg		2.10 (−1.40 to 5.60)	3.15 (−0.35 to 6.65)		−3.22 (28.82 to 22.39)	−16.96 (42.41–8.49)		−0.88 (−5.91 to 4.15)	0.78 (−4.42 to 5.97)		−2.33 (−8.46 to 3.81)	−1.11 (−7.37 to 5.16)	
> 3.5 kg		1.81 (−1.64 to 5.26)	1.13 (−2.34 to 4.60)		−16.41 (−38.44 to 5.61)	−6.63 (−28.71 to 15.46)		−7.43 (−12.66 to −2.20)	−2.43 (−7.83 to 2.96)		−6.87 (−13.06 to 0.68)	−2.96 (−9.42 to 3.49)	
Cord or lowest (pH)	22/55			0.42			0.74			0.72			0.91
pH < 7		2.92 (−0.57 to 6.42)	3.14 (−0.36 to 6.63)		−10.85 (−36.78 to 15.07)	−15.33 (−41.10 to 10.45)		−2.63 (−9.45 to 4.18)	−1.30 (−8.25 to 5.65)		−3.70 (−11.60 to 4.21)	−2.91 (−10.94 to 5.12)	
pH ≥ 7		−0.84 (−5.23 to 3.56)	1.06 (−3.37 to 5.49)		4.42 (−23.15 to 31.99)	−6.11 (−33.78 to 21.56)		−3.92 (−9.27 to 1.43)	1.07 (−4.70 to 6.84)		−3.30 (−9.80 to 3.21)	−0.67 (−7.52 to 6.19)	
Apgar Scores	26/65												
1 min				**0.02**			**0.01**			0.38			0.10
< 7		**2.33 (−0.16 to 4.81)**	**3.27 (0.77 to 5.76)**		**−11.96 (−29.02 to 5.10)**	**−19.89 (−36.93 to −2.85)**		−3.81 (−7.81 to 0.18)	−1.33 (−5.41 to 2.76)		−4.29 (−0.19 to 0.53)	−3.15 (−8.07 to 1.77)	
> 6		**−1.43 (−10.00 to 7.13)**	**−8.21 (−16.77 to 0.34)**		**15.32 (−46.99 to 77.62)**	**63.94 (1.71 to 126.17)**		−2.69 (−11.41 to 6.03)	6.59 (−4.09 to 17.28)		−4.56 (−12.72 to 3.60)	9.68 (−1.82 to 21.18)	
5 min	26/65			0.91			0.58			0.18			0.42
< 7		1.99 (−0.72 to 4.70)	2.49 (−0.24 to 5.23)		−13.64 (−30.68 to 3.41)	−17.07 (−34.18 to 0.05)		−5.27 (−9.49 to −1.04)	−1.63 (−5.88 to 2.63)		−5.13 (−10.22 to −0.05)	−3.43 (−8.61 to 1.75)	
> 6		2.02 (−3.56 to 7.59)	1.44 (−4.06 to 6.95)		3.45 (−41.68 to 48.58)	1.52 (−42.96 to 46.01)		2.27 (−5,18 to 9.73)	3.14 (−4.39 to 10.68)		−1.18 (−8.62 to 6.26)	3.35 (−4.74 to 11.44)	
10 min	26/65			0.61			0.45			0.49			0.85
< 7		3.05 (−0.03 to 6.13)	2.47 (−0.64 to 5.58)		−19.39 (−39.53 to 0.75)	−17.79 (−38.02 to 2.44)		−5.28 (−10.49 to −0.7)	−1.42 (−6.55 to 3.71)		−4.93 (−11.28 to 1.43)	−2.94 (−9.38 to 3.51)	
> 6		0.69 (−3.27 to 4.65)	1.93 (−2.01 to 5.87)		2.58 (−26.24 to 31.40)	−5.65 (−34.24 to 22.94)		−1.33 (−6.42 to 3.76)	0.61 (−4.66 to 5.87)		−3.18 (−8.67 to 2.30)	−0.41 (−6.15 to 5.33)	
HIE Grade	26/65			0.52			0.63			0.57			0.29
Mild		2.00 (−0.98 to 4.99)	3.05 (0.04 to 6.06)		−9.35 (−29.91 to 11.21)	−16.88 (−37.51 to 3.75)		−2.66 (−7.69 to 2.37)	−0.2 (−5.26 to 5.22)		−2.62 (−8.42 to 3.18)	−1.96 (−7.89 to 3.97)	
Moderate–severe		2.00 (−2.28 to 6.28)	0.30 (−3.95 to 4.55)		−9.72 (−40.86 to 21.41)	−1.68 (−32.42 to 29.06)		−5.62 (−10.87 to −0.37)	−1.28 (−6.27 to 3.70)		−7.63 (−14.37 to 0.89)	−1.40 (−8.01 to 5.21)	
Initial aEEG pattern	26/65			0.08			**0.04**			0.33			0.40
Normal		−1.99 (−11.26 to 7.28)	−6.47 (−15.78 to 2.84)		**23.33 (−46.70 to 93.36)**	**55.06 (−15.18 to 125.29)**		−1.33 (−17.36 to 14.69)	9.76 (−9.30 to 28.83)		1.32 (−14.51 to 17.14)	892 (−9.48 to 27.32)	
Abnormal		2.37 (−0.12 to 4.86)	3.09 (0.58 to 5.59)		**−12.75 (−29.83 to 4.32)**	**−19.00 (−36.04 to 1.96)**		−3.84 (−7.52 to −0.16)	−1.39 (−5.13 to 2.26)		−4.80 (−9.35 to −0.24)	−2.80 (−7.44 to 1.84)	
Age at cuddle	21/57			0.42			0.16			0.32			0.29
< 59 h		2.83 (−0.98 to 6.64)	4.01 (0.22 to 7.81)		−13.1 (−39.13 to 13.03)	−29.84 (−55.70 to −3.99)		−2.33 (−8.81 to 4.14)	1.51 (−8.06 to 5.03)		−3.10 (−10.06 to 3.86)	−31.6 (−10.14 to 3.82)	
≥ 59 h		1.70 (−1.87 to 5.27)	0.54 (−3.05 to 4.13)		−11.39 (−35.82 to 13.05)	1.47 (−23.04 to 25.97)		−7.21 (−12.50 to −1.91)	−0.26 (−5.78 to 5.27)		−8.87 (−15.60 to −2.14)	−1.24 (8.27 to 5.80)	
Inotropes	26/65			0.12			0.53			0.93			0.96
No		1.60 (−1.54 to 4.76)	3.47 (0.32–6.62)		−11.40 (−32.77 to 9.98)	−18.6 (−39.92 to 2.81)		−4.64 (−8.99 to −0.28)	−0.37 (−4.96 to 4.21)		−4.87 (−9.89 to 0.15)	−1.76 (−7.00 to 3.48)	
Yes		3.04 (−0.48 to 6.55)	−0.55 (−4.00–2.89)		−4.75 (−32.96 to 23.47)	2.51 (−25.18 to 30.20)		−2.27 (−8.83 to 4.29)	−0.77 (−7.48 to 5.95)		−3.40 (−11.76 to 4.96)	−2.11 (−10.64 to 6.43)	
Rewarming cuddles	26/65			0.99			0.94			0.89			0.71
No		1.88 (−4.12 to 7.88)	3.31 (−2.65 to 9.27)		−6.77 (−39.76 to 26.21)	−11.64 (−44.38 to 21.10)		−3.12 (−7.30 to 1.06)	−0.38 (−4.74 to 3.98)		−3.42 (−8.35 to 1.51)	−1.68 (−6.80 to 3.43)	
Yes		2.02 (−0.72 to 4.76)	2.21 (−0.53 to 4.95)		−9.75 (−29.49 to 9.98)	−13.23 (−32.97 to 6.51)		−6.84 (−14.72 to 1.04)	−2.00 (−10.25 to 6.24)		−8.35 (−18.23 to 1.53)	−2.93 (−13.19 to 7.33)	
Cuddle by	26/64			0.78			0.79			0.08			**0.04**
Mum		2.02 (−1.12 to 5.16)	1.73 (−1.31 to 4.87)		−8.22 (−30.03 to 13.59)	−8.65 (−3034 to 13.05)		−3.20 (−7.33 to 0.94)	2.50 (−2.02 to 7.03)		**−3.80 (−8.05 to 0.45)**	**1.96 (−2.72 to 6.65)**	
Dad		2.01 (−2.14 to 6.15)	3.36 (−0.82 to 7.53)		−13.56 (−42.39 to 15.27)	−21.22 (−50.16 to 7.71)		−7.14 (−15.92 to 1.64)	−7.88 (−16.74 to 0.98)		**−8.42 (−20.25 to 3.40)**	**−11.83 (−23.60 to −0.07)**	

*Note:* Values are marginal mean difference (95% CI). Summary values and statistical tests, derived from multi‐level model accounting for dependent data for infants and cuddles. Cord or lowest pH denotes the lowest pH from cord blood gas or blood gas taken from the infant within 60 min after birth. Significance for bold values are presented in the adjacent column that says *p*_interaction_.

Abbreviations: bpm, beats per minute; ms, milliseconds.

^a^ Numbers derived from log‐transformed values, and back‐transformed for presentation.

**FIGURE 2:**
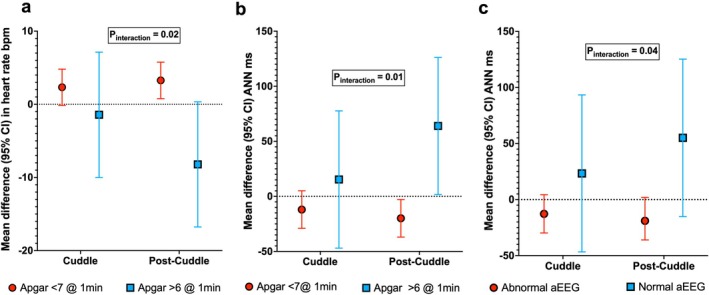
Mean difference (95% CI) in heart rate (a) and ANN (b) during and after cuddling, compared with pre‐cuddle of infants with Apgar score < 7 at 1 min, compared with infants with Apgar scores > 6 at 1 min. Mean difference (95% CI) in ANN (c) during and after cuddle compared with pre‐cuddle of infants with abnormal aEEG compared with infants with normal aEEG. bpm, beats per minute; ms, milliseconds.

There was evidence of an interaction between 1‐min Apgar score across the cuddle period for both heart rate (HR, *p*_interaction_ = 0.02) and ANN measures (*p*_interaction_ = 0.01) (Figure 2). Infants with a low 1‐min Apgar score demonstrated increases in heart rate during the cuddle (mean difference 2.33 bpm, 95% CI −0.16 to 4.81) and after the cuddle (3.27 bpm, 95% CI 0.77 to 5.76). In contrast, infants with a normal Apgar score showed reductions in HR during the cuddle (−1.43 bpm, 95% CI −10.00 to 7.13) and a larger decrease post‐cuddle (−8.21 bpm, 95% CI −16.77 to 0.34). Given an increase in heart rate would reduce the ANN, a similar interaction pattern was observed in the ANN. Infants with a low 1‐min Apgar score exhibited lower ANN values during the cuddle (−11.96 ms, 95% CI −29.02 to 5.10) and after the cuddle (−19.89 ms, 95% CI −36.93 to −2.85). Conversely, infants with a normal Apgar score showed increases in ANN during the cuddle (15.32 ms, 95% CI −46.99 to 77.62) and a marked increase post‐cuddle (63.94 ms, 95% CI 1.71 to 126.17).

A similar pattern was seen for the ANN measures between infants with a normal or abnormal aEEG pattern (*p*_interaction_ = 0.04), with infants with abnormal patterns showing lower ANN measures during (−12.75 ms, 95% CI −29.83 to 4.32), and after (−19.00 ms, 95% CI −36.04 to 1.96) the cuddle, in contrast to those with normal aEEG patterns (Cuddle: 23.33 ms, 95% CI −46.70 to 93.36), post‐Cuddle (55.06 ms, 95% CI −15.18 to 125.29) (shown in Figure 2). Further, there was an interaction between the parent who cuddled and the RMSSD values post‐cuddle, with the infant's RMSSD being significantly higher post‐cuddle compared with the pre‐cuddle values when cuddled by their mother (1.96 ms, 95% CI −2.72 to 6.65) than their father (−11.83 ms, 95% CI −23.60 to −0.07).

## 4 Discussion

In infants with HIE undergoing therapeutic hypothermia, CoolCuddle did not alter HRV at the group level, suggesting little evidence of adverse effect on autonomic regulation. However, infants with low 1‐min Apgar scores or moderate‐to‐severe encephalopathy exhibited distinct HRV and heart rate responses during and after cuddling compared with those with normal Apgar scores or mild encephalopathy. Furthermore, infants' RMSSD was significantly higher in the post‐cuddle period than in the pre‐cuddle period when cuddled by their mother compared with when cuddled by their father.

The absence of group‐level HRV changes indicates that CoolCuddle is physiologically stable and safe during therapeutic hypothermia. SDNN reflects overall heart rate variability and represents the beat‐to‐beat control mechanisms of both the sympathetic and parasympathetic nervous systems [21], while RMSSD and pNN50 primarily reflect parasympathetic activity [26]. In adults, higher HRV measures are associated with better cardiovascular health [27]. In preterm infants, HRV decreases during kangaroo care, possibly reflecting immature neural control [17]. In contrast, when kangaroo care was simulated using a mattress that mimicked the mother's breathing and heartbeats, there was no effect on the HRV measures, suggesting that skin‐to‐skin contact and co‐regulation with parents influence the autonomic regulation of infants [28]. Because CoolCuddle does not provide full skin‐to‐skin contact, its capacity to modulate autonomic nervous system activity may be limited.

At baseline, during the pre‐cuddle, infant characteristics were not associated with HRV measures, consistent with prior studies showing that HRV stabilises 24 h after birth in infants with HIE [29]. In our study, the mean age at which pre‐cuddle observation commenced was around 59 h after birth, and only one infant had the pre‐cuddle observation at 14 h after birth. Hence, we may not have observed any relationship between the infant characteristics and the HRV measures, which usually occurred within 24 h after birth. Our observation regarding the effect of asphyxia severity on HRV metrics concurred with other reports indicating that HRV metrics, including cardiac sympathetic index, normalised power in the high‐frequency band, and normalised power in the low‐frequency band, measured in the first 24 h or within 5 days after birth, were not associated with Apgar scores in infants with HIE [29].

Infants with lower Apgar scores exhibited higher heart rates and lower ANN during and after cuddling than during the pre‐cuddle period. In contrast, those with normal Apgar scores exhibited lower heart rates and higher ANN. Similarly, infants with moderate to severe HIE had lower ANN during and after cuddling than in the pre‐cuddle period, whereas infants with mild HIE had higher ANN during and after cuddling than in the pre‐cuddle period. This is consistent with Goulding et al.'s study, in which infants with moderate to severe HIE, compared with those with mild HIE, who received therapeutic hypothermia, had significantly lower SDNN, with the same direction of effect as ANN in our study [30]. However, we did not observe the impact of encephalopathy on HRV metrics during the pre‐cuddle. The differential HRV patterns observed in infants with more severe asphyxia or encephalopathy may reflect underlying neurological or cardiac dysfunction [31]. Although brainstem injury can disrupt HRV [32], none of the CoolCuddle participants had brainstem injury on their MRI, but during 34% of CoolCuddles, the aEEG were moderately or severely abnormal. We did not have echocardiography or cardiac troponin levels to determine whether infants who showed a differential response in HRV metrics during or after cuddling had cardiac dysfunction. During 34% of CoolCuddles, infants received inotropic support; however, receiving inotropic support is not a robust marker of cardiac dysfunction, as there would be variability in weaning the inotropes, and many infants receiving low‐dose inotropes may not have cardiac dysfunction.

We observed that the post‐cuddle RMSSD values were higher compared with the pre‐cuddle values when a mother cuddled their infant than their father did, implying an activation of the parasympathetic system. Although studies of preterm infants undergoing skin‐to‐skin contact with their parents show that skin‐to‐skin contact frequency is associated with increased parasympathetic system activation, as measured by HRV, the relationship between the parent who cuddles and HRV has not been elucidated [33]. There could be a confounding effect of morphine usage on HRV metrics, as morphine has been shown to reduce the low‐frequency component more than the high‐frequency component, suggesting dominance of the parasympathetic system [34].

Despite these limitations, our study has notable strengths, including a rigorous prospective design, continuous HRV monitoring across extended cuddle durations, and the use of each infant as their own control via multilevel modelling. Our findings demonstrate that Coolcuddle does not compromise autonomic stability and may be safely integrated into therapeutic hypothermia care pathways.

## 5 Conclusion

Autonomic regulation, as assessed by heart rate variability, remained stable throughout the CoolCuddle in infants with HIE undergoing therapeutic hypothermia. However, infants with low Apgar scores or with moderate to severe encephalopathy or depending on the parent who cuddled exhibited differential autonomic HRV and heart rate responses, possibly reflecting dysregulated autonomic function related to underlying neurological or cardiac impairment. These findings further reinforce the physiological safety of CoolCuddle and offer an opportunity for further studies examining parent‐infant co‐regulation in infants with HIE.

## Author Contributions

E.C.: conceptualisation, data curation, visualisation, validation, funding acquisition, investigation, methodology, project administration, resources, writing original draft, review and editing. S.O.: data curation, visualisation, validation, investigation, methodology, resources, review and editing. D.O.: data curation, visualisation, validation, data analysis, funding acquisition, investigation, methodology, writing, review and editing of manuscript.

## Funding

This work is independent research funded by the National Institute for Health Research (NIHR), Research for Patient Benefit research programme grant number PB‐PG‐1217‐20020. The funder had no role in the design, data collection, data analysis and reporting of this study.

## Ethics Statement

This study protocol was reviewed and approved by Research Ethics Committee Northern Ireland [19/NI/0143] and the UK Health Regulatory Agency [IRAS ID 257430].

## Consent

We obtained written informed consent from all participants' legal guardians.

## Conflicts of Interest

The authors declare no conflicts of interest.

## Supporting information


**Table S1:** Absolute value and relative changes of heart rate variability measures during the CoolCuddle.

## Data Availability

All summaries of individual data measures are presented in this article and its Supporting Information. Further enquiries regarding the raw data are available from the corresponding author upon reasonable request.
