# Supplementary material for: CoolCuddle and Autonomic Regulation in Infants With Hypoxic‐Ischaemic Encephalopathy: An Intervention Study
Source: Acta Paediatr. 2026 Apr 16;115(8):1688–99. doi: 10.1111/apa.70547 (PMC13371796; doi:10.1111/apa.70547)
Supplement: Supplementary file 1 — Table S1: Absolute value and relative changes of heart rate variability measures during the CoolCuddle. [file APA-115-1688-s001.docx]

**Supplementary Table 1.** Absolute value and relative changes of heart rate variability measures during the CoolCuddle

| Variable | Infants/Cuddles | Pre-cuddle | During cuddle | Post cuddle | P value$ |
| --- | --- | --- | --- | --- | --- |
| NN50 | 26/65 |  |  |  |  |
| Summary measures |  | 10.53 (1.888) | 9.59 (1.70) | 11.93 (2.13) |  |
| Mean Difference (95% CI) |  | Ref (0) | -0.94 (-3.50 to 1.61) | 1.40 (-1.50 to 4.29) | 0.92 |
| pNN50 | 26/65 |  |  |  |  |
| Summary measures |  | 6.30 (0.80) | 6.44 (0.81) | 6.96 (0.89) |  |
| Mean Difference (95% CI) |  | Ref (0) | 0.15 (-1.21 (-1.21 to 1.50) | 0.67 (-0.77 to 2.11) | 0.94 |

Values are marginal mean (SE), or marginal mean difference (95% CI) as appropriate

Summary values, and statistical tests, derived from multi-level model accounting for dependent data for infants and cuddles

$ p-value derived from the likelihood ratio test; comparing models with, or without the (fixed effect) cuddle-period variable.

Numbers derived from log-transformed values, and back transformed back for presentation
